# Different Traits Determine Introduction, Naturalization and Invasion Success In Woody Plants: Proteaceae as a Test Case

**DOI:** 10.1371/journal.pone.0075078

**Published:** 2013-09-24

**Authors:** Desika Moodley, Sjirk Geerts, David M. Richardson, John R. U. Wilson

**Affiliations:** 1 Centre for Invasion Biology, Department of Botany and Zoology, Stellenbosch University, Stellenbosch, South Africa; 2 Invasive Species Programme, South African National Biodiversity Institute, Kirstenbosch Research Centre, Cape Town, South Africa; University of Rome ‘La Sapienza’, Italy

## Abstract

A major aim of invasion ecology is to identify characteristics of successful invaders. However, most plant groups studied in detail (e.g. pines and acacias) have a high percentage of invasive taxa. Here we examine the global introduction history and invasion ecology of Proteaceae—a large plant family with many taxa that have been widely disseminated by humans, but with few known invaders. To do this we compiled a global list of species and used boosted regression tree models to assess which factors are important in determining the status of a species (not introduced, introduced, naturalized or invasive).

At least 402 of 1674 known species (24%) have been moved by humans out of their native ranges, 58 species (14%) have become naturalized but not invasive, and 8 species (2%) are invasive. The probability of naturalization was greatest for species with large native ranges, low susceptibility to *Phytophthora* root-rot fungus, large mammal-dispersed seeds, and with the capacity to resprout. The probability of naturalized species becoming invasive was greatest for species with large native ranges, those used as barrier plants, tall species, species with small seeds, and serotinous species.

The traits driving invasiveness of Proteaceae were similar to those for acacias and pines. However, while some traits showed a consistent influence at introduction, naturalization and invasion, others appear to be influential at one stage only, and some have contrasting effects at different stages. Trait-based analyses therefore need to consider different invasion stages separately. On their own, these observations provide little predictive power for risk assessment, but when the causative mechanisms are understood (e.g. *Phytophthora* susceptibility) they provide valuable insights. As such there is considerable value in seeking the correlates and mechanisms underlying invasions for particular taxonomic or functional groups.

## Introduction

Species introduced to areas outside their natural dispersal ranges need to overcome various barriers to establish, persist, proliferate and spread [1], [2]. Because some invasive species present a major threat to global biodiversity [3] it is important to understand the full suite of drivers of invasion to mitigate species impacts and prioritize management efforts.

Identifying traits correlated with invasiveness is a central goal in invasion ecology, the success of which has direct application for the prediction and prevention of future invasions [4]. Although consistent determinants of plant invasiveness are elusive, several general predictors have emerged [5], [6], [7]. Across a large range of plant taxa invasive species tend to have short juvenile periods, short reproduction intervals, small seed masses and large native range sizes [8], [9], [10]. Such generalizations are based on associations identified between particular traits and the position of species along the introduction-naturalization-invasion (INI) continuum, and where possible are linked to the underlying mechanisms [9], [11], [12], [13], [14], [15]. Such studies are important because introduced species are influenced by different factors at each stage of the INI continuum and these interacting factors and processes determine the fate of introduced species [2], [16]. However, taxonomic groups vary markedly in the proportion of species that are invasive [17] and most groups systematically studied, with respect to invasive traits, have many invasive taxa.

Among woody plants, pines (genus *Pinus* L.) and Australian acacias (*sensu* [18]) have been proposed as model groups for elucidating the determinants of invasiveness [18], [19]. These taxa contain many species, have a long history of introduction to many parts of the world, and contain many species at different stages of the INI continuum [8], [20], [21], [22]. We argue that Proteaceae is an excellent group to test whether findings in these model groups are generally applicable. Unlike for *Pinus* and Australian acacias, Proteaceae species have been moved primarily for flower production or horticulture, though like the two model groups many species have had a long history of introduction [23]. Despite the large number of introduced species in Proteaceae, only a few species have become invasive, but the interest in Proteaceae for the production of cut flowers and in other forms of horticulture is increasing in many parts of the world [24], and so it is important to understand the determinants of invasiveness in the group to prevent future invasions.

Proteaceae is a large family of flowering plants occurring predominantly in the Southern Hemisphere with its greatest diversity in Australia and southern Africa [25], [26], [27]. The family is typically associated with nutrient-poor soils and many species have adaptations for surviving in these conditions, such as proteoid roots [28], [29]. Species of Proteaceae should thus, arguably, be more likely to establish in novel environments than species of pines or acacias which are reliant on mycorrhizal or rhizobial mutualists to facilitate growth [30]. Many Proteaceae are also serotinous, having woody follicles that open after fires to release seeds into low-competition environments [31], facilitating establishment and spread.

The horticultural trade is an important pathway for introducing invasive alien plants [32], [33], [34]. Therefore, one would expect there to be several invasive Proteaceae species since many species are cultivated as horticultural plants which contributes to a high propagule pressure and therefore a high probability of establishment. The main genera used for floriculture are *Banksia* L.f., *Leucadendron* R.Br., *Leucospermum* R.Br. and *Protea* L.. Species in the genera *Aulax* Berg., *Grevillea* R.Br. ex Knight, *Isopogon* R.Br. ex Knight, *Mimetes* Salisb., *Paranomus* Salisb., *Serruria* Salisb. and *Telopea* R.Br. are used to a lesser extent [35]. In addition to ornamental uses, species of *Grevillea*, *Hakea* Schrad. & J.C.Wendl., and *Macadamia* F.Muell are grown for food production, as barrier plants or windbreaks, and as landscape plants.

In this study, we aimed to identify whether the factors underlying invasion success identified for model groups like pines and Australian acacias apply to another less frequently invasive group of woody plants. Specifically we ask: which Proteaceae have been introduced worldwide? what is the invasion status of these introduced species? are there any taxonomic biases in invasion? is there a general set of traits associated with naturalization or invasion that are similar to findings from research on acacias and pines, or are such traits specific to Proteaceae?

## Methods

### Global proteaceae inventory

We developed a global list of Proteaceae species from many sources (listed in Table S1 and S2). We based the number of genera in this family according to the list compiled by Weston and Barker (80 genera and 1702 species; [25]), updated with several recent changes, e.g. the merging of *Banksia* and *Dryandra* ([36]; see Table S2a for reference to the complete species list). Synonyms were taken into account during searches and name changes were documented (See Table S2b for more details).

### Status as introduced species

We conducted extensive surveys of databases, floras, published sources and corresponded with experts (for lists of sources consulted see Table S1) to develop lists of the status of species along the INI continuum. Species were recorded as introduced if they were recorded from a biogeographical region outside their native range, naturalized if they were known to form self-replacing populations, and invasive if they formed self-replacing populations at considerable distances from parent plants and showed the potential to spread over long distances (i.e.>100 m in <50 yrs for taxa spreading by propagules; discussion in [37]). Species were only recorded as naturalized or invasive if this was clearly mentioned in the literature or when this could be confirmed through communication with experts.

### Taxonomic patterns

To assess how invasiveness differs across the genera of Proteaceae, we plotted the proportion of species in each genus that were of a particular status (introduced, naturalized, or invasive) against the species richness of that genus. We generated a random expectation for how the proportion of species of a particular status should change with genus size using the hypergeometric distribution [38]. Genera falling between the 95% confidence intervals (after correcting for multiple comparisons using the false detection rate test (p.adjust in R)) were considered similar to that of a random expectation. Genera above or below the intervals were significantly over- or underrepresented respectively.

### Selection of traits

We collated species trait information (Table 1; see Table S3 for reference sources used) for traits that have been shown to be useful for predicting invasiveness in previous studies [15], [21], [39], [40], [41], [42]. In addition, because Proteaceae species are mainly introduced for horticulture, we assessed whether inflorescence size and use (i.e. purpose for species introductions) are important for promoting the likelihood of introduction.

**Table 1:** Traits used in the analyses for separating introduced vs. naturalized and naturalized vs. invasive Proteaceae species.

Trait	Methods of measuring	No. of species in the full dataset	No. of Australian species	Categories
Inflorescence size	Horticultural trait: Small inflorescences (<100 mm in width or length) coded 0; large inflorescences (≥100 mm in width or length) coded as 1	359	200	Categorical, binary
Use	Horticultural trait: Agro-forestry, barrier plants, ornamental plants, forestry, fuel, land rehabilitation. Species used for tanning and medicinal purposes were not included in these groups, since we found no confirmation during surveys that these species were introduced specifically for these purposes	352	196	Categorical
Height (m)	Maximum height reported in literature	365 (0.1–40; 2.5)	202 (0.1–40; 3)	Continuous
Life-form	Based on whether species were reported as trees or shrubs	369	207	Categorical
Maturity	The number of years a species takes to first flowering	181 (1–9; 2)	28 (1–9; 3.5)	Continuous
Flowering duration	The number of months in a year that species are in flower (calculated from the start and end of flowering months)	366 (1–12; 4)	204 (1–12; 4)	Continuous
Regeneration mechanism	Species regeneration method: re-seeder coded 1; resprouter coded 0.	343	187	Categorical, binary
Serotiny	Seeds retained on the plant coded 1, non-serotinous (i.e. stored in the soil) coded 0	357	195	Categorical, binary
Dispersal	Vector of seed dispersal: Unspecialized dispersal, wind, water, mammals, ants and birds	309	154	Categorical
Bird pollinated	Pollination primarily by birds coded 1; pollination by other vectors coded 0	305	150	Categorical
Compatibility	Self-compatible coded 1; self-incompatible coded 0	114	39	Categorical, binary
Range size (km^2^)	Total area a species occupies in its natural range calculated using minimum convex polygons	375 (2–3516000; 29190)	204 (131–3516000; 82360)	Continuous
*Phytophthora*	Degree of susceptibility to root rot fungus. Resistant (Res): unaffected species; susceptible (Sus): diseased plants with a lower chance of death; & very susceptible (VS): plant death	120	81	Categorical
Seed mass (g)	Seed weight reported in the database	197 (2.02–504.70; 19.34)	100 (2.74–501.80; 20.17)	Continuous

The range and median values for continuous variables are shown in parentheses.

### Analysis of traits important at various stages

To explore the relationship between the explanatory variables (i.e. the traits listed in Table 1) and response variables (i.e. the status of species, Table S4) we used boosted regression trees (BRT). This is a machine learning approach which builds a multitude of simple tree models independently and then produces one combined model based on their predictive performance [43]. This method makes use of two powerful techniques, boosting and regression trees [43]. The boosting component of this method increases the predictive performance of the model and reduces over-fitting [43]. BRT models generate an index of relative influence of all variables; this is calculated by summing the contribution of each variable. This meant we could assess the importance of factors at each stage in the invasion process using the relative influence of the explanatory variables and the amount of variance explained by the model. All analyses were carried out in R (version 2.15.1, R Development Core Team, 2012) using the gbm package for BRT [44].

As few trait data are available for species that have not been introduced, and the availability of these data are likely to be from a biased subset of species, we only constructed two BRT models, introduced (but not yet naturalized) vs. naturalized; and naturalized (but not yet invasive) vs. invasive. Before constructing the BRT models we tested for co-linearity between the predictor variables using the Kendall rank correlation coefficient. Since there was no strong correlation in the data between any two variables (max r^2^ = 0.64, Figure S1) we included all variables in the analyses. BRT models were then fitted with Bernoulli error distributions since the response variables are binary (naturalization model: 0 =  introduced (but not yet naturalized) species and 1 = naturalized species; invasion model: 0 = naturalized (but not yet invasive) species and 1 = invasive species).

For each stage, we selected the optimum model settings based on recent guidelines [43]. We specifically aimed to achieve a model with at least 1000 trees with minimum predictive deviance. Height, seed mass and range size were log transformed for the analyses. The results are plotted as fitted functions which represents the effect of each predictor variable on the response variable, while taking into account the effect of all other variables in the model [43]. The fitted BRT naturalization and invasion models comprised the following parameter settings: a two-way interaction model (tree complexity = 2) with a slow learning rate of 0.0005 and a bag fraction of 0.5. Tree complexity limits the number of nodes allowed for each tree in the boosting sequence to main effects only (tree complexity = 1) or interaction of variables (e.g. tree complexity = 2); the learning rate specifies the weight of each successive tree added to the prediction model; and the bag fraction parameter specifies the proportion of data selected at each iteration which improves predictive performance [43]. The final models comprised an optimal number of 2600 trees for the naturalization model, while the loss function was minimized at 5500 trees for the invasion model.

We initially performed the analysis using the full dataset comprising 14 predictor variables (Table 1). The model showed native range size to be one of the important variables determining naturalization (Figure S2). Since most of the naturalized species and all invasive species are from Australia, and native range sizes differed for the different bio-geographic regions, we decided to restrict the rest of the analysis to Australian taxa (Table S5). This also allowed the inclusion of the range sizes of non-introduced species as this data is available for almost all Australian taxa (Atlas of Living Australia's online database, http://www.ala.org.au., accessed June 2012; and the Global Biodiversity Information facility database, http://data.gbif.org, accessed June 2012). To test the importance of range size along the INI continuum we used independent Mann-Whitney Wilcoxon tests.

## Results

**Figure 1:**
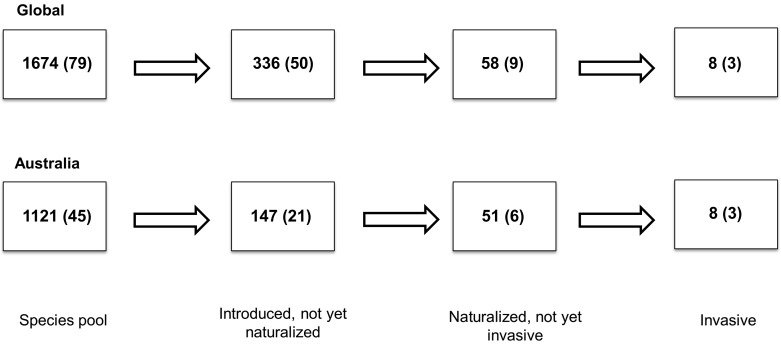
The number of Proteaceae species that are introduced, naturalized or invasive. Out of the 1674 species in the family at least 402 species have been introduced worldwide. Out of the 402 species, 336 species have not yet naturalized, 58 species are naturalized but not recorded as invasive and 8 species are invasive. In the same manner, out of the 1121 Australian species at least 206 species have been introduced, of which 147 have not yet naturalized, 51 are naturalized but not invasive and 8 are invasive. Numbers of genera in each category are shown in parentheses.

At least 402 species (24%) out of the 1674 species recognized here have been introduced outside their native ranges (Figure 1; Table S4). Out of these 402 species introduced globally, 336 species (84%) have not yet naturalized, 58 species (14%) are considered naturalized, but not invasive, and 8 species (2%) are invasive. Australia is home to 1121 Proteaceae species and at least 206 species (18%) are known to have been introduced to other regions (Figure 1; Table S4). Out of these 206 species, we recorded 147 Australian species (71%) that have been introduced out of their native range but which have not yet naturalized, 51 naturalized species (25%) which are not yet invasive, and 8 invasive species (4%). All invasive species and ∼90% of the naturalized species are native to Australia, and all invasive species are present in South Africa.

**Figure 2:**
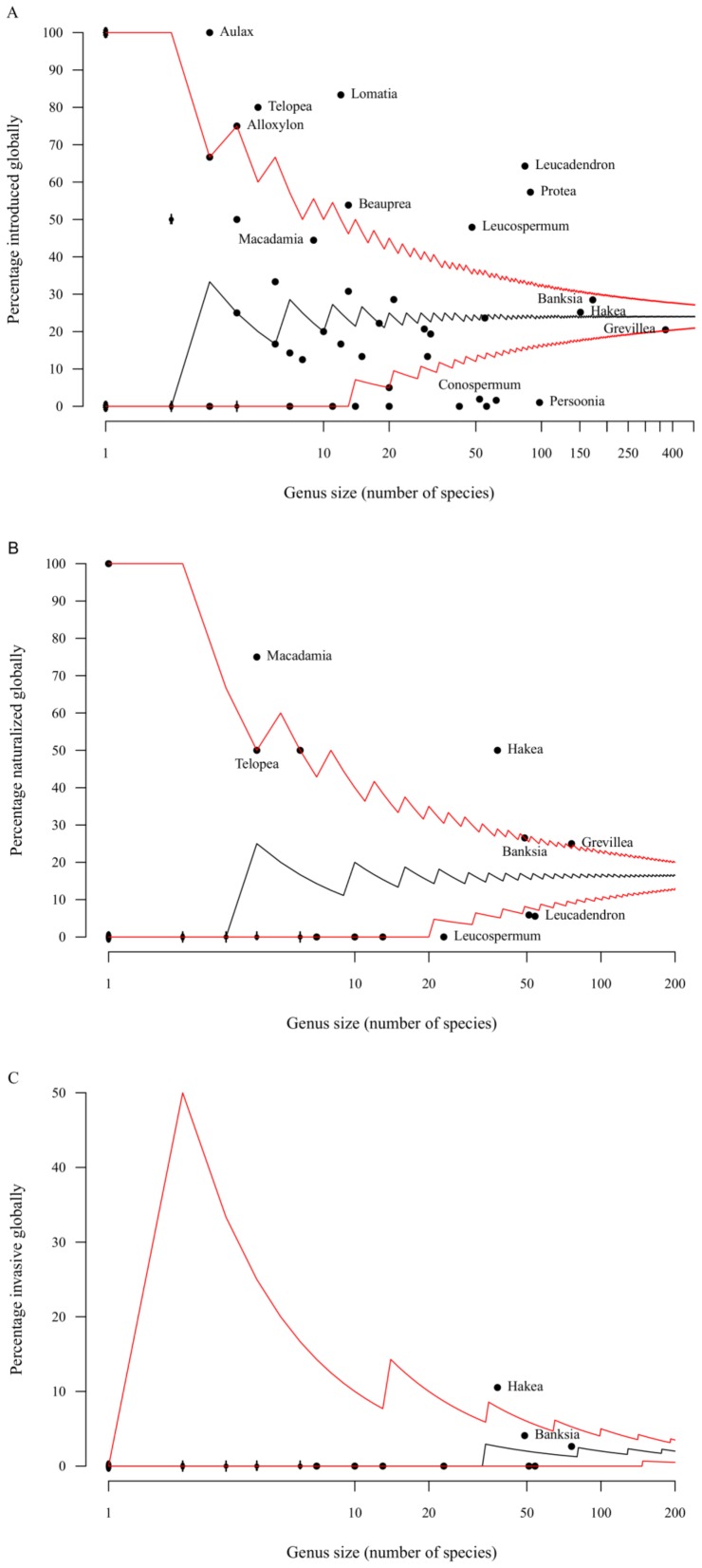
Taxonomic distribution of Proteaceae genera worldwide. Patterns depict A) introduced, B) naturalized and C) invasive species. Each point represents a genus (to avoid clutter only selected genus names are included) with lines indicating expectations from a hypergeometric distribution (median and 95% confidence intervals). Genera falling between the lines are not significantly over- or underrepresented. Genera above or below the intervals are significantly over- or underrepresented respectively. To assess how invasiveness differs across the genera of Proteaceae.

Of the 79 Proteaceae genera, most have a similar number of naturalized or invasive species to that expected from a random distribution (Figure 2), but eight genera are over-represented and seven are under-represented in the list of introduced Proteaceae (Figure 2A). Moreover, 29 genera contain species which have naturalized, with three Australian genera (*Macadamia*, *Hakea* and *Grevillea*) over-represented on the lists and three South African genera (*Leucadendron*, *Leucospermum* and *Protea*) under-represented (Figure 2B). *Hakea* is the only genus over-represented in terms of invaders (Figure 2C).

### Transition from introduction to naturalization for Australian Proteaceae

The BRT naturalization model accounted for 12% of the mean total deviance (1-mean residual deviance/mean total deviance). The six most influential variables predicting naturalization of Australian species are native range size, dispersal vectors, susceptibility to *Phytophthora*, fire regeneration mechanisms, seed mass and the number of flowering months (Table 2; Figure S2).

**Table 2:** Summary of the boosted regression tree models of factors associated with naturalization (a) and invasion (b) in Proteaceae species.

Trait	Percentage contribution	Range of fitted values (min, max)	Description of effect
**a) Naturalization**			
Range size	26.5	−1.30, −0.63	Species with larger native ranges are more likely to naturalize (Figure 3A)
Dispersal	18.4	−1.22, −0.89	Although wind dispersal is the most common, species that are dispersed by mammals tend to naturalize (Table S6)
*Phytophthora*	16.5	−1.41, −0.80	Less susceptible species are more likely to naturalize (Figure 3B)
Regeneration mechanism	11.6	−1.17, −0.96	Species that survive fires by resprouting are more likely to naturalize
Seed mass	8.2	−1.18, −0.98	Species with larger seed sizes are more likely to naturalize
Flowering duration	6.3	−1.20, −1.06	Species flowering over longer periods are more likely to naturalize
**b) Invasion**			
Barrier	33.4	−2.79, −1.42	Barriers plants are more likely to invade (Figure 3C)
Height	22.1	−2.76, −1.62	Taller species are more likely to invade (Figure 3D)
Range size	16.1	−2.56, −1.92	Species with larger native ranges are more likely to invade (Figure 3A)
Serotiny	8	−2.49, −2.03	Species with canopy-stored seed banks are more likely to invade
Seed mass	8	−2.38, −2.11	Species with small seeds are more likely to invade
Regeneration mechanism	6.3	−2.50, −2.08	Species that regenerate from seed are more likely to invade

**Figure 3:**
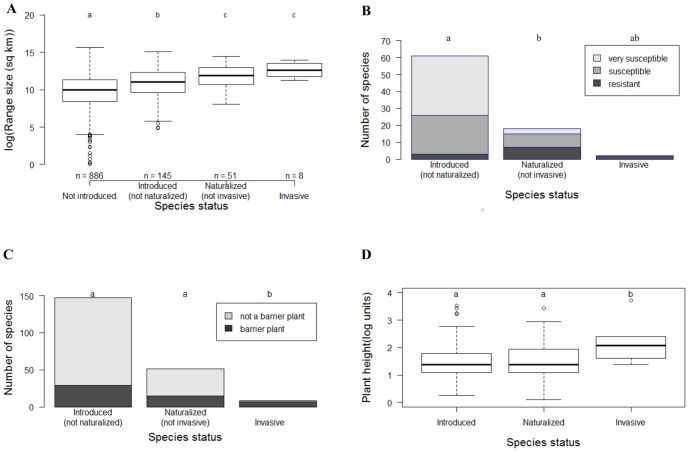
Factors associated with introduction, naturalization, and invasion in Australian Proteaceae species. A) native range size; B) the number of susceptible and resistant species to *Phytophthora*; C) use as barrier plants; and D) plant height (m). Different letters indicate groups that differed significantly at *p*<0.05. For barrier plants and susceptibility to *Phytophthora*, Fisher's exact test for count data was used. Only factors that explained at least 15% of either model are shown.

Only traits contributing at least 5% to the models are shown; traits that explained at least 15% of either model are shown in Figure 3 and Table S6. Data range includes the minimum and maximum values from the fitted functions and is representative of effect size.

### Transition from naturalization to invasion for Australian Proteaceae

The BRT invasion model accounted for 36% of the mean total deviance. Barrier plants, plant height, native range size, seed mass, serotiny and fire regeneration mechanisms comprised the six most influential variables predicting invasion (Table 2; Figure S3).

### Influential variables predicted from the BRT models

The source pool of 1121 Australian species encompasses a large geographic distribution. Native range size differed significantly across stages in the invasion continuum (Figure 3A). Introduced species had larger native ranges than non-introduced species (*W* = 55874, *p*<0.05, 95%CI = −59378 to −30957), naturalized species had larger ranges than the pool of introduced species (*W* = 2954, *p*<0.001, 95%CI = −146446 to −25971), and, although invasive species had the largest native ranges on average (447688 km^2^±136193, mean ± SE), they did not have significantly different native range sizes compared to naturalized species (*W* = 136, *p* = 0.13, 95%CI = −370985 to 61624).

Several other variables were important. The level of *Phytophthora* susceptibility prominently influences naturalization success (Figure S2). Only a few susceptible species managed to survive and establish, and, although not significant, only resistant species progressed to become invasive (Figure 3B).

Species response to fire differed between the stages of invasion. Resprouters were more likely to become naturalized (Figure S2) but re-seeders (serotinous species) had a greater chance of becoming invasive (Figure S3).

Seed mass was an important predictor of naturalization and invasion, but in contrasting ways. For naturalization, large seeds (34.48 g±5.79) are important (Figure S2). Conversely, small seeded plants (23.21 g±3.47) are more likely to invade (Figure S3). Dispersal vector was also important for naturalization. Species dispersed by mammals are more likely to naturalize and wind dispersal also comprises an important vector for a large proportion of species (Figure S2; Table S6).

Species that flowered for longer periods had a higher probability of successfully naturalizing (Figure S2). The length of a long flowering period varied from four months to all year round.

Australian Proteaceae species have been introduced worldwide for many uses, but the pool of introduced species mainly comprised species used as barrier plants and for ornamental purposes (Table S4). Many introduced species have multiple uses. For example, *Banksia ericifolia* is used for ornamental purposes, as a barrier plant and for cut flowers. The BRT invasion model predicted the use as barrier plants to be the most important trait conferring invasiveness (Figure 3C; Figure S3).

Finally, plant height is an important correlate of invasiveness for Proteaceae, with tall species having a significantly (*W* = 108, *p* = 0.03) higher tendency to become invasive (Figure 3D).

## Discussion

We found analysing the Proteaceae family a useful exercise for testing emerging generalizations in plant invasion ecology. Specifically this study revealed that: a) human usage determines the extent to which different Proteaceae genera are introduced, but a range of traits are associated with the different stages of the invasion process; b) some traits (e.g. native range size) show effects consistent with those seen in model groups; c) for some traits there was a clear mechanism for the association of the trait to invasion success (e.g. the level of susceptibility to *Phytophthora*); d) some traits show differing responses at the different stages of invasion (e.g. seed mass); and e) some traits are linked to the context in which a species is planted (e.g. barrier plants), which creates greater opportunities for invasion rather than directly affecting population growth rate or spread rate.

Within Proteaceae, unsurprisingly, species that are useful to humans have been introduced more often than those with less obvious attractiveness or utility, e.g. genera with showy flowers are overrepresented in the introduced species (e.g. *Protea*, Figure 2A). Once the introduction barrier is overcome, different variables are important for naturalization and invasion, and the genera that are overrepresented tend to be those used for food production or as barrier plants (e.g. *Hakea* and *Macadamia*; Figure 2B). Therefore, we see a taxonomic bias of species that are attractive and useful to humans being introduced worldwide.

The effect of native range size is similar to that seen in many other taxonomic groups [9], [10], [41]. Proteaceae species with large native ranges are more likely to be introduced, naturalize, and become invasive (Figure 3A). There are a few potential explanations for this. Firstly, humans are more likely to encounter widespread species and introduce them to other regions [45]. Secondly, wide-ranging species are inherently more tolerant of a wider range of environmental conditions than species with smaller ranges - increasing their probability of becoming established in a new area [45]. Lastly, species with larger ranges can more easily be matched to suitable climates prior to introduction to ensure successful establishment.

The mechanistic explanation is much clearer for susceptibility to *Phytophthora*. Few very susceptible species have naturalized, and only resistant species are invasive (Figure 3B). A number of *Phytophthora* species are known to affect Proteaceae, the most common being *P. cinnamomi* and *P. nicotianae* [46]. These fungi cause a range of effects, notably root-rot, which often kill infected plants [46]. Resistant species therefore require more attention due to their risk of becoming naturalized and invasive, while highly susceptible species pose a very low invasion risk (though might be equally hard to cultivate).

For both *Phytophthora* susceptibility and native range size the direction of the effect was the same at both the naturalization and invasion stages, but this was not the case for other traits. Plant height was only found to influence one stage (i.e. invasion success (Figure 3D)). Plant height has been shown to be correlated with invasiveness in many studies [5]. Many Proteaceae species have wind-dispersed seeds, tall plants can therefore disperse their seeds further. This will increase spread rates, and therefore also invasiveness, but will not necessarily increase the rate of successful naturalization.

In contrast, seed size influenced both naturalization and invasion, but the direction of the effect differed. Large-seeded species had a higher chance of becoming naturalized, whereas small-seeded plants were more likely to invade. The success of naturalization could be due to large seeds having greater nutrient reserves favouring establishment. But if a species can establish, small-seeds are beneficial for long-distance dispersal and therefore favour invasive spread [47]. Seed size is known to be an important determinant of invasiveness [8], [48], and these findings are similar to other plant groups where large seed size promotes the growth of introduced species and small seed size favours successful invasions [11], [49].

Similarly, we found resprouters were more likely to naturalize while reseeders were more likely to invade. Vegetative reproduction has been shown to be a common predictor of invasiveness [6], [50], [51]. Species that reproduce vegetatively are ideal for cut flowers and hedges because they are tolerant to heavy harvesting. These plants need to allocate resources into coppicing and thus less into fruit production (i.e. low propagule pressure). Because of their smaller propagule pressure resprouting plants can take longer to spread, but they will likely be more persistent and harder to control [52]. The observed trend could merely be an artefact of recent introductions (i.e. resprouters require more time to progress along the INI barriers) and is therefore only recognized as important for naturalization in Proteaceae and not invasion. All eight invasive species were either introduced for cut flowers, as ornamentals, or as hedge/barrier plants. These uses require plants to be nurtured from the time of planting which possibly explains how reseeders overcame the initial survival barriers and naturalized. Regeneration from seeds is an ideal mechanism for driving invasions when recruitment events are favourable [52]. Proteaceae tend to occur in fire-prone environments. Therefore, an investment in producing seeds rather than allocating resources to vegetative reproduction will be more advantageous in environments with short fire-return intervals, such as in fynbos. Fire regimes potentially explain why introduced South African Proteaceae (83% of which are reseeding species, Table S4) have failed to invade Australian ecosystems (long fire-return intervals delay recruitment) whereas introduced Australian serotinous species have been so successful in South African fynbos (short fire-return intervals provide favourable conditions for recruitment and dispersal of seeds). In addition, reseeders produce higher seed loads which increases propagule pressure and thus the likelihood of invasion [52]. Propagule pressure, widely shown to be a major determinant of naturalization and invasion success [53], [54], is also important for successful invasion of Proteaceae species.

Finally, one of the most striking results was that all but one of the invasive species are used as barrier plants – although less than a quarter (24.7%) of introduced *Proteaceae* are used mainly for this purpose (Figure 3C). We believe this is due to an interaction between the location of plantings and the occurrence of fires. Plants in gardens and orchards (80.1% are planted for ornamental purposes) are generally protected from fires and because many species are serotinous, there is limited release of seeds in the absence of fires. Other uses (e.g. species planted for forestry, fuel and land rehabilitation) expose plants to fires, but these factors were not predicted as important determinants of invasiveness probably because there are just a few species introduced for these purposes. Species used as barriers or hedges are typically planted on the edge of farms or homesteads and in some cases adjacent to natural vegetation. These land-use practices often increase the risk of spread [55]. For example, *Banksia ericifolia* when cultivated for flower production showed signs of naturalizing but not spreading. However, these cultivated plantings were protected from fire. When plantings are exposed to natural fire regimes, the species can spread quickly and invade natural vegetation [56]. These observations support predictions that, based on its life history, *B. ericifolia* was a high-risk alien plant in the region [57], [58], suggesting there is considerable value in conducting trait-based assessments for specific groups [59]. This example also illustrates that invasion success is determined by several factors; species traits (e.g. serotinous woody follicles), habitat characteristics (e.g. an area that burns regularly) and the context in which a species is planted (e.g. hedge plant). Therefore, a combination of traits must be taken into account when seeking to explain invasion success or failure.

## Conclusions

There are already a few serious Proteaceae invaders, but since many species have only recently been widely planted, there are potentially many more major invaders “waiting in the wings”. The traits correlated with Proteaceae introductions and invasion highlight intriguing similarities as well as differences between invasion stages and with the generalizations drawn from other model groups. Therefore, to gain a better insight on the determinants driving invasions we need to examine each invasion stage separately since it is likely that different traits will become important across different stages of the invasion process. On their own, these observations provide little predictive power for risk assessment, but when the causative mechanisms are understood valuable management insights can be drawn. By understanding which traits are correlated to introduction, naturalization and invasion success; what the mechanisms behind such correlations are; and under which conditions invasions are favoured we can go a long way to providing accurate predictive risk assessments.

## Supporting Information

Figure S1 **Correlation (r) tests between all predictor variables using the global dataset.**
(PDF)

Figure S2 **Plots of fitted functions for each term in the BRT naturalization model.** This model only includes species native to Australia. Fitted functions depict the effect of each predictor variable after accounting for the effects of the other predictors in the model. Plots are ordered by the contribution of each variable, in parentheses.
(DOCX)

Figure S3 **Plots of fitted functions for each term in the BRT invasion model.** This model only includes species native to Australia. Plots are ordered by the contribution of each variable, in parentheses.
(DOCX)

Table S1 **The furthest point along the introduction-naturalization-invasion continuum that Proteaceae species are recorded as having reached using different datasets.**
(XLS)

Table S2 **Reference list of species names and synonyms.**
(XLS)

Table S3 **Seventy-four literature sources and online databases that were used, in combination, to collate information on the explanatory variables.**
(XLS)

Table S4 **Raw data of all introduced, naturalized and invasive species and the fourteen traits that were measured. See** **Table 1** **for metadata.**
(XLS)

Table S5 **Significance of native range size (km^2^) in the linear regression model fitted to species regions of origin.** Native range size differed significantly between Australia and other regions of origin.
(XLS)
