# Supplementary material for: Different Traits Determine Introduction, Naturalization and Invasion Success In Woody Plants: Proteaceae as a Test Case
Source: PLoS One. 2013 Sep 24;8(9):e75078. doi: 10.1371/journal.pone.0075078 (PMC3782508; doi:10.1371/journal.pone.0075078)
Supplement: Figure S2 — Plots of fitted functions for each term in the BRT naturalization model. This model only includes species native to Australia. Fitted functions depict the effect of each predictor variable after accounting for the effects of the other predictors in the model. Plots are ordered by the contribution of each variable, in parentheses. (DOCX) [file pone.0075078.s002.docx]

**Figure S2**. **Plots of fitted functions for each term in the BRT naturalization model**. This model only includes species native to Australia. Fitted functions depict the effect of each predictor variable after accounting for the effects of the other predictors in the model. Plots are ordered by the contribution of each variable, in parentheses.
