# Supplementary material for: Different Traits Determine Introduction, Naturalization and Invasion Success In Woody Plants: Proteaceae as a Test Case
Source: PLoS One. 2013 Sep 24;8(9):e75078. doi: 10.1371/journal.pone.0075078 (PMC3782508; doi:10.1371/journal.pone.0075078)
Supplement: Figure S3 — Plots of fitted functions for each term in the BRT invasion model. This model only includes species native to Australia. Plots are ordered by the contribution of each variable, in parentheses. (DOCX) [file pone.0075078.s003.docx]

**Figure S3**. **Plots of fitted functions for each term in the BRT invasion model. This model only includes species native to Australia**. Plots are ordered by the contribution of each variable, in parentheses.
