# Supplementary material for: Different Traits Determine Introduction, Naturalization and Invasion Success In Woody Plants: Proteaceae as a Test Case
Source: PLoS One. 2013 Sep 24;8(9):e75078. doi: 10.1371/journal.pone.0075078 (PMC3782508; doi:10.1371/journal.pone.0075078)

Figure S1. Correlation (r) tests between all predictor variables using the global dataset.

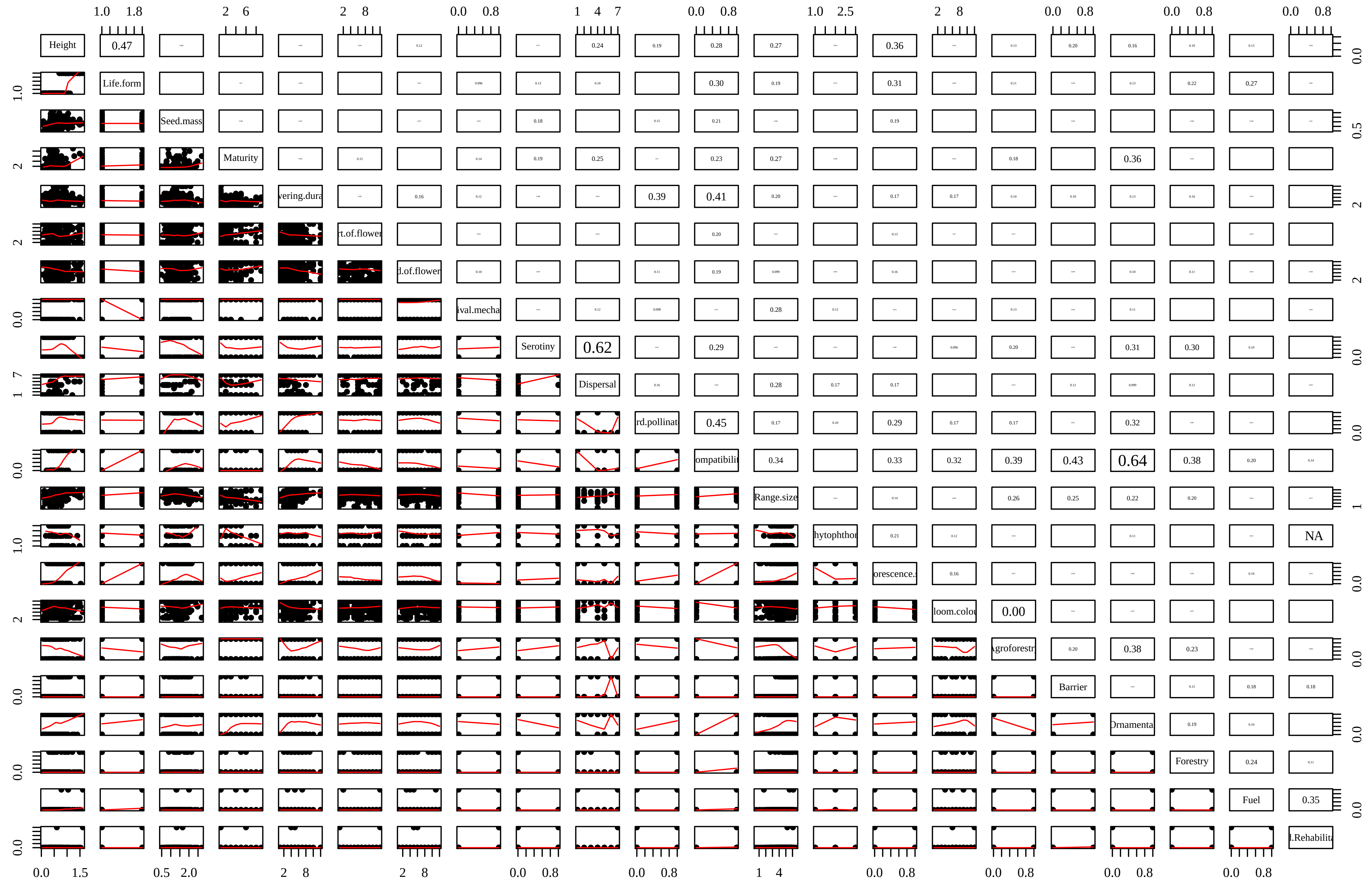

Supplement: Figure S1 — Correlation (r) tests between all predictor variables using the global dataset. (PDF) [file pone.0075078.s001.pdf]
